# Traumatic Cardiac Arrest—A Narrative Review

**DOI:** 10.3390/jcm13020302

**Published:** 2024-01-05

**Authors:** Patrick Schober, Georgios F. Giannakopoulos, Carolien S. E. Bulte, Lothar A. Schwarte

**Affiliations:** 1 Department of Anesthesiology, Amsterdam University Medical Center, 1081 HV Amsterdam, The Netherlands; p.schober@amsterdamumc.nl (P.S.);; 2 Helicopter Emergency Medical Service‚ Lifeliner 1, 1044 AN Amsterdam, The Netherlands; 3 Department of Surgery, Amsterdam University Medical Center, 1105 AZ Amsterdam, The Netherlands

**Keywords:** traumatic, cardiac, arrest, hypovolemia, hemorrhage, oxygenation, tension, pneumothorax, tamponade, resuscitation

## Abstract

A paradigm shift in traumatic cardiac arrest (TCA) perception switched the traditional belief of futility of TCA resuscitation to a more optimistic perspective, at least in selected cases. The goal of TCA resuscitation is to rapidly and aggressively treat the common potentially reversible causes of TCA. Advances in diagnostics and therapy in TCA are ongoing; however, they are not always translating into improved outcomes. Further research is needed to improve outcome in this often young and previously healthy patient population.

## 1. Introduction

In the treatment of traumatic cardiac arrest (TCA) a paradigm shift occurred in recent decades, switching resuscitation in TCA from a merely futile attempt to a more promising intervention [1]. Several reasons account for this, ranging from a better understanding of the pathophysiology of TCA, division into distinct TCA entities [2], separation from other causes of CA, refined guidelines [3], and also improved training [4] on this formerly potentially neglected topic [5]. In addition, technical advances in both diagnostics (e.g., point of care ultrasound, POCUS) and therapy [6] further reshape the management of TCA. Thus, although overall mortality of TCA is still very high [7], outcomes in selected subgroups are improving. This review of the broad topic of TCA will, with the inherent limitations of a review, summarize our current understanding of TCA key points.

## 2. Epidemiology

Regarding demographics in TCA, a recent Swedish study reviewed almost 300 adult TCAs (~2/3 from blunt trauma) in a Level I trauma center. TCA patients were on average relatively young (~40 years), mostly males (~80%), and relatively healthy (~60%) before TCA [1]. Age and previous health status support recovery potential of the patients, but also underline the economically and societal burden of productive life years lost in unsalvageable cases of TCA. Even in unsalvageable cases, the young age and previously healthy condition may support the successful transfer of those patients into organ donor programs [8,9]. Epidemiological differences exist between continents and countries. For example, the widespread availability of firearms in the United States does impact the epidemiology of TCA in the US [10], compared to most European countries [5].

### Etiology

The etiology of TCA may be grossly divided into potentially reversible causes and non-reversible causes, e.g., extensive irreparable damage to vital organs. Often, it is challenging to make this diagnostic decision already upon arrival of the patient in the trauma center; thus, therapeutic interventions are regularly initiated in patients with in hindsight non-reversible causes of TCA. Termination of TCA therapy in those cases must not be considered as failure of the trauma team but rather as an ethical appropriate decision withholding valuable resources in a futile situation of medically senseless activism.

**Figure 1:**
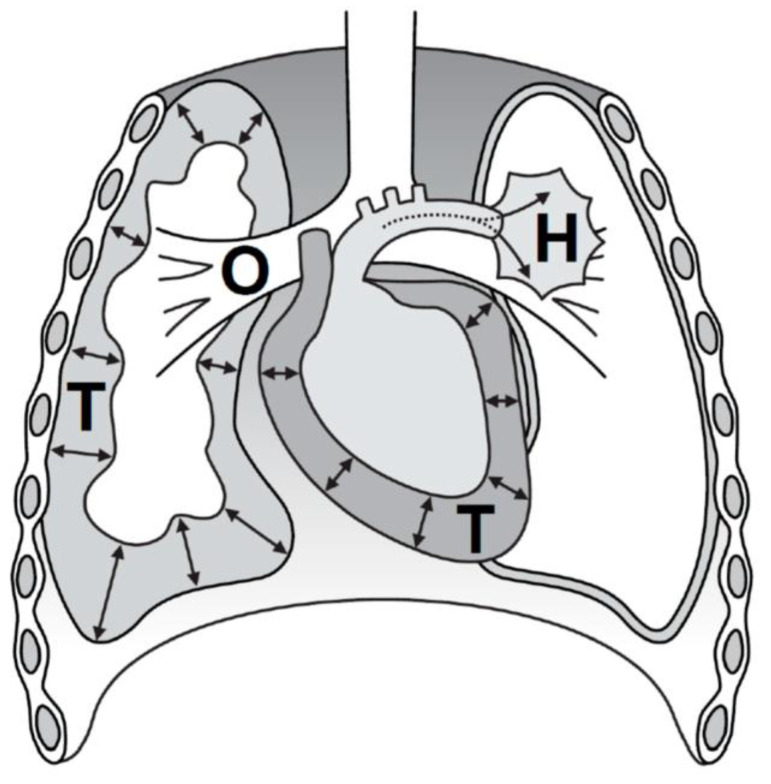
Common reversible causes of traumatic cardiac arrest.

The focus of TCA treatment is to rapidly address the potentially reversible causes of TCA; however, so far there is no universally accepted approach to cover this. One memory aid is the acronym H.O.T.T. (Figure 1), e.g., endorsed by the European Resuscitation Council (ERC), explained as follows:

The figure depicts the most common, potentially reversible causes of traumatic cardiac arrest (TCA), made memorable by the H.O.T.T. acronym: Hypovolemia (H) in trauma is almost exclusively caused by hemorrhage and is the most common, potentially reversible cause of TCA. Impairment of oxygenation (O) often requires the creation of a patent airway and optimizing oxygen supply. A tension pneumothorax (T) may present in spontaneously breathing patients, but also rapidly develop after endotracheal intubation and mechanical ventilation. A pericardial tamponade (T) may both be caused by blunt or penetrating chest trauma. Addressing all H.O.T.T. items rapidly is a key concept in current TCA treatment.

- **H**ypovolemia (**H**emorrhage, until proven otherwise);
- **O**xygenation impairment;
- **T**ension pneumothorax;
- **T**amponade of the pericardium, i.e., ‘cardiac tamponade’.

The acronym H.O.T.T. resembles a trauma-related subset from the ALS-memory-aid ‘4xH and 4xT’ for all common reversible causes of cardiac arrest (i.e., 4xH represent hypovolemia, hypoxemia, hyper/hypokalemia, hypothermia and 4xT represent thrombosis, toxins, tamponade and tension pneumothorax). The individual H.O.T.T. etiologies can occur in combination, and can aggravate each other. The distribution of these H.O.T.T. etiologies are for hypovolemia 48%, impaired oxygenation 13%, tension pneumothorax 13% and pericardial tamponade 10% [11].

## 3. Outcomes

The traditional paradigm of futility of TCA resuscitation has shifted within recent decades towards a more optimistic perspective on TCA outcomes, particularly in selected subgroups of patients. However, reviewing the outcome of TCA is hampered by differences in the included study populations, e.g., including either all TCA patients, or only initially resuscitated TCA patients, or only TCA patients transported to the hospital or the ICU [12]. Owing to these differences in inclusion criteria, survival to discharge may vastly vary from ~4% to ~40% [12]. One study from the United Kingdom reports an overall 30-day survival of 7.5% in 705 TCA patients, with even a 11.5% survival, when TCA was successfully resuscitated already in the prehospital phase [13].

In the military setting, published survival data range from 0% (Israel armed forces, 0/149 survivors until hospital admission [14]), to more optimistic results of 11% (of 424) [15]. Particularly in TCA cases where Return of Spontaneous Circulation (ROSC) can rapidly be achieved, neurological outcome appears to be better than in other causes of cardiac arrest [16,17]. Immediate resuscitative efforts in TCA focusses on simultaneous treatment of potentially reversible causes, which take priority over sole chest compressions or early adrenaline administration. The response to TCA thus is time-critical and success depends on a well-established chain of survival, including advanced pre-hospital and specialized trauma center care.

In pediatric TCA, survival figures are at least comparable and thus also in children, resuscitation from TCA is not per se futile exercise [16], however, survival does not equal beneficial neurological outcome even with the higher plasticity of children’s brains.

Individual prognosis of outcome after TCA is difficult to predict, but certain factors are commonly associated with improved outcome [12]. Beneficial demographic factors include younger age and female sex. Regarding mechanism of injury and physiology, improved outcomes are associated with extremity injuries, a lower injury severity score, shockable ECG rhythms, and reactive pupils. Regarding treatment and treatment logistics, improved outcomes are reported for witnessed TCA, bystander CPR, short transport times, and certain interventions, such as airway management [12].

In a more general approach, prognostic factors for TCA survival have been systematically reviewed and extracted [18] from almost 40.000 TCAs from 53 studies. Although some traditionally outcome-associated factors of TCA remained herein unproven (e.g., differences between blunt and penetrating trauma), two factors associated with improved outcomes were the presence of a shockable cardiac rhythm (e.g., ventricular tachycardia), and cardiac motion on ultrasound. Asystole, thus a non-shockable rhythm with no cardiac motion has, just as in cardiac arrest from medical causes, the worst prognosis. Also, PEA without cardiac motion on POCUS has an extremely poor prognosis, as detailed below.

### 3.1. Diagnostic Dilemma of PEA versus Pseudo PEA

The initial diagnosis of TCA is usually made clinically by attesting unconsciousness, absent or agonal spontaneous respiration and absence of the central palpatory pulses in a patient with evident or suspected adequate trauma [3].

However, a TCA with complete cessation of blood flow cannot always be distinguished from a traumatic peri-arrest, both in the prehospital setting or immediately upon arrival in the trauma bay. For example, a PEA as confirmed by absence of a palpable central pulse may still reveal blood flow, e.g., when cross checked by point of care ultrasound (POCUS) [19], hence termed ‘pseudo PEA’. A ‘pseudo PEA’ is thus a state of severe hypotension with blood pressure below the subjective and notoriously unreliable threshold of manual palpatory pulse detection [19]. In contrast to asystole and a ‘true’ PEA, the group of pseudo PEA are a subset of patients with comparably good prognosis, particularly if the cause such as profound hypovolemia is rapidly treated. However, maltreated pseudo PEA is likely to deteriorate into full cardiac arrest. On the other hand, treating a pseudo PEA as true PEA may also be detrimental, e.g., by performing chest compressions [20] and by administering 1 mg adrenaline injections to a patient with pseudo PEA, e.g., just caused by severe, but otherwise simple-to-treat hypovolemia.

The association between POCUS findings and patient outcomes from TCA has been systematically reviewed, reporting no survival case to hospital discharge in the absence of cardiac motion, whereas 7% survival to discharge was observed with POCUS-confirmed cardiac motion [21]. However, the authors do not promote a change in TCA guidelines based on these data, since the patient numbers included were too small. Others note, since PEA without cardiac motion on POCUS has an extremely poor prognosis, it may, just like asystole, be a reason to consider terminating resuscitation in TCA rapidly after potentially reversible causes have been addressed.

Thus, in the management of TCA, the role of POCUS is still developing. The merits of POCUS have been demonstrated in other fields, but a critical view on POCUS is still warranted particularly in TCA settings with numerous possible pitfalls of POCUS [22]. Furthermore, POCUS must only be performed if the results are expected to change treatment, thus adding ‘need to know’ (e.g., cardiac tamponade) versus ‘nice to know’ (e.g., exact location of a liver laceration) information [23,24,25].

In respect to PEA and pseudo-PEA, the literature is not always uniform on defining the component of “electrical activity”. Although usually some form of organized electrical activity is meant, some sources demand an ECG-complex frequency of >20 bpm, whereas others do not.

### 3.2. Therapy

Although the traditional ABCD-system (Airway, Breathing, Circulation, Disability) is not primarily developed for patients in TCA, the trauma team members are usually task-distributed in this fashion (e.g., see ATLS or ETC courses) and thus the ABCD-system may be used to address the H.O.T.T. entities in a rearranged fashion: hypovolemia resembles a C-problem, oxygenation impairment an A/B-problem, tension pneumothorax a B/C-problem, and cardiac tamponade again a C-problem. Ideally, these potentially reversible causes of TCA should be addressed simultaneously rather than sequentially if resources and personnel allow.

‘Airway’ challenges: In TCA the airway problems are frequently functional, e.g., airway obstruction by a sagging tongue, soft palate or epiglottis in unconscious or arrested victims. In addition, airway obstruction may be caused by gastric regurgitation, blood, or other materials [26]. However, less frequently, airway problems in TCA can be anatomical, e.g., with extensive maxillofacial injuries or direct trauma to the airway [27]. In a subset of those cases, the airway problems are the primary cause of TCA, e.g., by airway obstruction in patients with extensive trauma to the anterior neck.

Generally, functional airway problems are relatively easy to manage with standard airway maneuvers (e.g., jaw trust) and simple tools (e.g., oropharyngeal airway, suction devices), whereas anatomical airway problems can be challenging to handle and may require expert support (e.g., an anesthesiologist) and specialized equipment (e.g., surgical airway sets) [27,28].

In the initial phase of TCA treatment it is pivotal to create an open (patent) airway for (re-)oxygenation, but not necessarily to create an secured airway (i.e., by a cuffed endotracheal tube). Thus, deliberately de-emphasize or postpone time- and resource-intense airway-securing maneuvers, e.g., endotracheal intubation, in the very initial phase of resuscitation, when an open, patent airway is present or can quickly be established non-invasively.

Also in a peri-arrest setting, delaying intubation saves critical resources (personnel, time) and limits the detrimental cardiovascular effects of drug-assisted intubations and subsequent positive pressure ventilation, particularly in under-resuscitated, hypovolemic patients. This is in line with a ‘resuscitation before intubation’ approach. Studies indicate the detrimental effects of a traditional ABC approach with early endotracheal intubation, on hemodynamics and outcome in critical trauma patients [29].

Furthermore, intubations in trauma patients are hampered by anatomical (e.g., facial trauma) and functional (e.g., rapid desaturation, sensitivity to anesthetics) patient-related factors, but also non-patient-related factors, such as crew resource management (CRM) aspects. Thus, intubation attempts in trauma patients may contribute to TCA [30].

‘Breathing’ challenges: Potentially reversible causes of TCA include a tension pneumothorax [5,31] and less frequently a massive hemothorax. Less common causes of TCA include an entero-thorax after traumatic diaphragmatic rupture, multiple rib fractures (flail chest), or aspiration of blood, all potentially causing TCA by impairment of breathing and oxygenation. Since oxygenation may be impaired on several levels of the oxygen cascade in TCA and traumatic peri-arrest, and measurements such as SpO_2_ and blood gas analysis are not available initially in TCA settings, administering 100% high flow oxygen to all TCA patients is advocated.

Positive pressure ventilation may aggravate hypotension and hypoperfusion by impeding venous return to the heart, particularly in hypovolemic patients. Low tidal volumes, minimal PEEP levels, and slow respiratory rates may help optimize cardiac preload. Ventilation should always be monitored with continuous waveform capnography and adjusted to achieve target end tidal CO_2_ levels after ROSC.

### 3.3. Tension Pneumothorax

**Figure 2:**
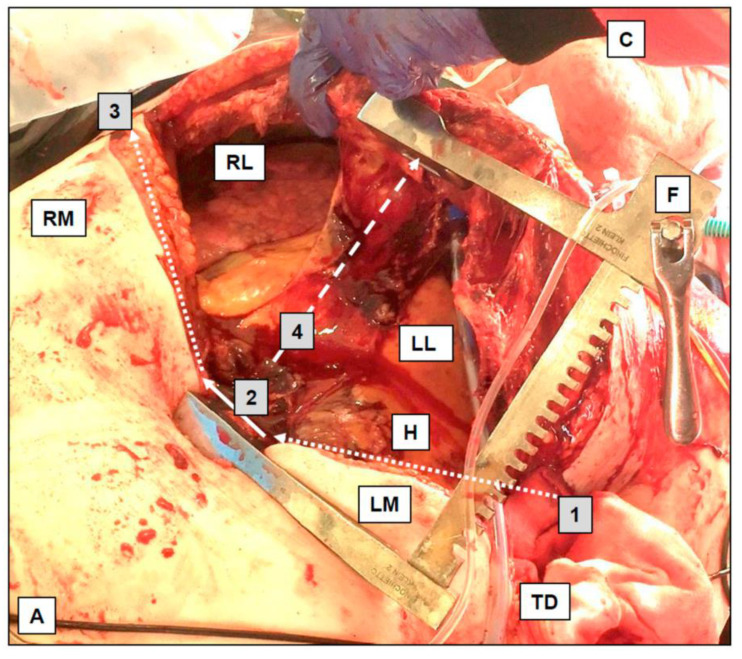
Resuscitative thoracotomy (‘clamshell thoracotomy’).

A tension pneumothorax is present in 13% of all TCA cases. Immediate chest decompression is indicated upon suspicion, and some sources suggest a bilateral routine chest decompression in patients with TCA and no other immediate treatment priorities. Scalpel-based thoracostomies or mini thoracotomies, often finger-assisted (‘finger thoracostomy’), are disputably more effective than ordinary needle thoracocentesis [32], and are faster than conventional chest tube insertion. The thoracostomies are performed in the 4th or 5th intercostal space (ICS), just anterior to the mid-axillary line. The same approach is applied with a suspected critical hemothorax or hemopneumothorax. In selected cases, thoracostomies may be extended to a resuscitative thoracotomy (i.e., clamshell thoracotomy, exemplified in Figure 2) [6].

This figure depicts a typical aspect after a resuscitative thoracotomy, i.e., clamshell thoracotomy, with key aspects of the surgical access to the intrathoracic organs. Starting with a thoracostomy in the fourth or fifth intercostal space (here: on the left side), the scalpel incision is extended towards the left margin of the sternum (1, dotted line, here above left mammilla, LM). The sternum is horizontally dissected (2, by a strong scissor or Gigli-type wire saw, solid arrow). The sternal dissection is extended on the right side towards the right mid-axillary line (3, dotted arrow, here above the right mammilla, RM). The sternum is then distracted with a Finochietto (F) rib-spreader (4, dashed line) to achieve the ‘clamshell’ situation, allowing access to the intrathoracic structures (LL = left lung; RL = right lung; H = heart; C = chin; A = abdomen; TD = initially placed thoracic drain). A resuscitative thoracotomy is both diagnostic and therapeutic. The bilateral thoracotomy decompresses possible tension pneumothoraxes and hemothoraxes. Intrathoracic access allows hemorrhage control with the application of direct pressure or vessel clamping. The pericardium is opened to evacuate a possible tamponade and to facilitate internal cardiac compressions. Cardiac injuries can be addressed with staplers, sutures, or balloon obstruction (e.g., by a Foley catheter). Compression or cross-clamping of the descending thoracic aorta facilitates the redistribution of cardiac output towards the brain and heart, whilst simultaneously limiting distal hemorrhage, e.g., from abdominal or pelvic vessels.

Thoracic needle decompressions are less effective, but still appropriate [33] to buy time if scalpels or the required expertise for thoracostomies are not available, and may still be appropriate in children. Preferred needle location in children is the second ICS in the mid clavicular line, and the fourth or fifth ICS just anterior to the mid axillary line in adults. Based on studies, 45 mm needles appear too short in many adults [34], and longer needles (e.g., 70 mm [35]) should be available. However, whether decompression needle type and needle insertion location have relevance for patient outcome, remains questionable [36]. In addition, needle decompression does have typical risks, e.g., cardiac injury and cardiac tamponade [37]; thus, training is mandatory. Since (tension-)pneumothoraxes in trauma patients are often associated with a hemothorax, needle-based catheter techniques are prone to clotting and obstruction of the small bore cannula and thus must be frequently rechecked.

TCA patients may present with a tension pneumothorax or it may rapidly develop during TCA resuscitation, shifting treatment priorities during resuscitation. For example, in a hypoxic patient, placing an endotracheal tube and mechanical ventilation might be the appropriate lifesaving intervention. However, initiation of mechanical ventilation may rapidly convert a rather harmless, even undetected pneumothorax into a life-threatening tension pneumothorax, becoming a secondary trigger of TCA itself, necessitating chest decompression as the now immediate lifesaving intervention.

## 4. ‘Circulation’ Challenges

### 4.1. Hypovolemia Causing TCA

The most common potentially reversible cause of TCA is hypovolemia, mostly by exsanguination, i.e., in ~50% of TCA cases. Given this high a priori probability of hypovolemia as a cause or contributor to TCA, assuming hypovolemia until proven otherwise is a reasonable strategy in TCA. Although rapid and aggressive fluid infusion, or, increasingly, blood (-product) transfusion, may potentially reverse TCA at least temporarily, the underlying source of hemorrhage must be addressed as quickly as possible (fix the leaking tank and refill the tank). Major hemorrhage may present as external bleeding, usually already addressed in the field (e.g., by tourniquet placement), or as internal bleeding. Internal localizations of major hemorrhage include the body cavities (thorax, abdomen, pelvis) and fractured major bones. Treatment options for TCA with internal hemorrhage range from pelvic binder placement to invasive techniques such as the REBOA or resuscitative thoracotomy with aortic cross-clamping. The indications and benefits of certain interventions are not universally accepted yet; e.g., the role of emergency department REBOA [38] and resuscitative thoracotomy in TCA remains debatable [39,40].

In the peri-arrest setting, during impeding TCA or after an arrest has been resolved by ROSC, blood pressure management is challenging [41]. Generally, in the TCA peri-arrest setting it appears reasonable to adopt the management concept of ‘damage control resuscitation’, i.e., including permissive hypotension, resuscitative coagulation management, and damage control interventions [42,43]. However, although permissive hypotension herein is an promising concept to limit further blood loss [44], particularly patients with traumatic brain injury may require higher blood pressures, compared to other trauma patient categories [45]. Thus peri-arrest blood pressure management in the context of TCA is challenging, poses dilemmas, and should be individualized based on patient and trauma characteristics [46,47].

### 4.2. Cardiac Rhythms in TCA

Asystole (~40%) and PEA (~25%) are the prevalent ECG rhythms in TCA, according to a Swedish study [1]. In contrast, shockable rhythms such as VF were rare (only ~7%), but associated with a better prognosis [1]. Although the traditional mantra of immediate, high-quality CPR [20] with no (or minimal) pauses is de-emphasized in traumatic versus medical cardiac arrest; shockable rhythms should be shocked also in TCA immediately. According to a meta-analysis, initial shockable rhythms occur in TCA in about 6.8% [18]. Practitioners should be aware that a shockable rhythm also in patients with trauma may indicate a medical rather than traumatic etiology of the arrest and must also be treated as such.

### 4.3. Chest Compression in TCA

The role of chest compressions in TCA is controversial. Chest compressions, both manual and device assisted, are likely less effective in hypovolemic subjects. Based on this, the European Resuscitation Council and others promote a ‘Don’t pump an empty heart’ directive. This concept is not yet proven in high-quality human studies, but large animal studies demonstrate that in hypovolemic low flow states, chest compression may not only be ineffective, but even harmful, e.g., in hemorrhaged pigs [48], dogs [49], or baboons [50].

In addition to (hemorrhagic) hypovolemia, chest compression in obstructive TCA etiologies, e.g., tension pneumothorax and cardiac tamponade, are likely also less effective [12,50]. Considering the economics of resuscitation, resources (e.g., time and personnel) may be allocated to more beneficial, high-yield interventions, instead of performing ineffective or even harmful chest compressions.

Therefore, chest compressions should take a lower priority than immediate treatment of reversible causes in TCA, e.g., controlling hemorrhage. Studies deprioritizing chest compressions through the implementation of a novel regional paramedic TCA protocol (Victoria state, Australia) to prioritize rapid treatment of reversible TCA causes did, however, not demonstrate a survival benefit yet [51], and further research is needed.

However, if in doubt, and in certain subgroups of TCA, high-quality chest compressions should be performed. These cases include possible medical or cardiac causes of arrest, but also traumatic cardiac arrest from non-hypovolemic, non-obstructive etiologies, such as isolated TBI, cardiac contusion, or asphyxia [52].

### 4.4. Cardiac Injuries Causing TCA

A subgroup of cardiac injuries presents with potentially reversible patho-mechanisms of TCA, both penetrating and blunt cardiac injuries. Typical examples include relatively simple cardiac injuries, such as a single stab wound of the heart, complicated by pericardial tamponade. If pericardial tamponade caused TCA, rapid pericardial decompression may achieve ROSC alone, or with additional measures (e.g., temporary cardiac massage). Pericardial tamponade may not only follow penetrating trauma of the ‘cardiac box’ or ‘danger box’, but may also result from blunt impact of the chest. Obviously, aside pericardial decompression, also the primary cardiac injury should be fixed at least temporarily.

Cardiac tamponade is the underlying cause of approximately 10% of cardiac arrest in trauma. One typical indication for resuscitative thoracotomy (RT) is cardiac tamponade. Where there is traumatic cardiac arrest and penetrating trauma to the chest or epigastrium, immediate RT (e.g., via a clamshell incision) can be lifesaving. The chance of survival is about four times higher in cardiac stab wounds than in gunshot wounds. As with resuscitation of TCA, also the traditional dogma of ‘futility of emergency thoracotomies’ has to be abandoned, certainly for indications such a single thoracic stab wounds. Studies demonstrate survival rates from severe trauma-related emergency thoracotomies from 7% in blunt trauma [53] to more than 50% [54] in not yet arrested patients.

In a patient with cardiac tamponade and still a pulse present, rapid transfer to the operation theatre for surgery may be a reasonable strategy. However, if the patient is in arrest, an immediate RT in de emergency department is a promising strategy. Usage of traditional improvised or commercial tamponade percutaneous needle-based kits have several downsides, including misplacement and in-effectivity (e.g., clotting of the catheter) and should therefore be avoided, if RT options are available.

### 4.5. Cardiac Contusion

Cardiac contusion after high-energy thoracic impact may cause TCA [55]. Publicly known cases occur in sports, e.g., football [56,57], but are also relevant in the non-sport setting [58]. Resuscitation of TCA after cardiac contusion primarily follows A(C)LS guidelines, with rapid initiation of high-quality CPR, including early defibrillation upon indication, as the main components of resuscitation. However, cardiac contusion may also follow traffic accidents, e.g., thoracic impact of a vehicle driver against a steering wheel. In those cases, TCA may be complicated by other thoracic injuries, e.g., blunt aortic injury.

### 4.6. Cardiac Electrotrauma-Induced Arrest

Injury by electric currents can cause TCA, classically by inducing cardiac arrhythmias, even without anatomical cardiac damage. In electro-induced arrhythmias, such as VF, standard ALS algorithms apply for resuscitation.

‘Disability’ challenges: A loss of cerebral function, e.g., a loss of consciousness in TCA, may result from cerebral trauma itself, or from extra cerebral processes, e.g., loss of consciousness after cerebral hypoperfusion in cardiac arrest following other causes.

Resuscitation of TCA complicated by traumatic brain injury (TBI) has traditionally been regarded futile; however, a retrospective study supports a more optimistic perspective [59]. This study demonstrated hospital discharge in 8/42 patients, with 7/42 patients even discharged with favorable neurologic outcomes [59].

TCA may occur after (isolated) TBI, even without major structural brain damage. Herein, a specific cause is coined ‘impact brain apnea’ [60]. Patients with ’impact brain apnea’ may present with a surprisingly good outcome after TCA if treated adequately in the early phase. These patients often later reveal diffuse axonal injury (DAI) or (posthypoxic) brain edema in imaging, but not necessarily with major structural brain lesions. In contrast to many other patients with TCA, these patients are basically treated according to the ‘standard’ guidelines of (medical) cardiac arrest, with a focus on (re-)oxygenation and high-quality chest compression.

Spinal injuries, particularly affecting the cranial spinal segments C3/C4/C5, which feed the phrenic nerve, can cause insufficient breathing, respiratory arrest, and ultimately TCA. Secondary injury to the spinal cord should be prevented by spinal immobilization if feasible, however, in TCA settings, treatment of reversible causes and achievement of ROSC has priority over resource-intense cervical spine protection (‘life before limb’).

Severe impairment of the nervous system leading to central apnea and TCA can also be induced by electro injuries.

The above covered challenges of ‘Airway’, ‘Breathing’, ‘Circulation’ and ‘Disability’ require specific therapeutic and diagnostic skill sets. A skill increasingly applied for several diagnostic questions and also (re-)evaluation after therapeutic interventions is POCUS, both in and outside the hospital [61,62,63]. Several protocols have been developed to apply POCUS systematically in critical trauma patients including TCA, e.g., a modified FATE-, RUSH-, or eFAST protocol [64,65]. Performed systematically, POCUS will detect major reversible causes of TCA, e.g., hypovolemia, tension pneumothorax, or cardiac tamponade, and prompt respective interventions, such as a resuscitative thoracotomy.

## 5. Guidelines

### 5.1. Foundation of TCA Guidelines

Prospective high-quality studies, i.e., RCTs, on TCA to inform guideline development, are virtually absent, despite the large impact of the TCA population on the patient, health care, and society. Therefore, TCA treatment guidelines are often based on a mix of expert opinions (e.g., Delphi procedures), retrospective studies, or extrapolated from related medical fields [3]. This mix of informing sources partly explains why guidelines for treatment of TCA of several organizations show considerable differences, e.g., when comparing national [66] or international guidelines, e.g., between Advanced Trauma Life Support (ATLS) or European Resuscitation Council (ERC) and the European Trauma Course (ETC) [3].

### 5.2. Guidelines, When to (Not) Initiate TCA Resuscitation

TCA resuscitation may be initiated when there are no obvious signs of futility of resuscitation [67] and, currently, there is no universal consensus under which criteria resuscitation in TCA should (not) be initiated [3]. The following criteria to withhold TCA resuscitation are published in the literature; however, they are difficult to follow in practice, e.g., because of the notorious uncertainty of reported time points and other information. The American College of Surgeons and National Association of Emergency Physicians suggested to withhold resuscitation attempts in TCA when death is inevitable, but also in trauma cases with apnea, pulselessness and absence of an organized ECG [67]. The ERC considers the following criteria to withhold or terminate resuscitation in TCA [3].

Withhold resuscitation, if

- No signs of life for at least 15 min;
- Catastrophic injuries, e.g., penetrating head injury, loss of brain tissue.

Consider terminating resuscitation, if

- No ROSC after potentially reversible TCA causes were addressed;
- No cardiac motion in POCUS, even with organized ECG activity present, after the reversible causes of TCA were addressed.

Prolonged resuscitation efforts in futile TCA cases unnecessarily increase risks for the direct care providers and bystanders for stick or cut-injuries, infections, psychological trauma, and drain-limited system resources (e.g., personnel, ER capacity, and blood products). In cases where the typical reversible causes of TCA have been ruled out, or addressed, termination of treatment may be considered directly [3]. However, others suggest continuing with an additional period (e.g., 10 min) of conventional CPR as a reasonable, practical decision, although this period is not backed up by high-quality data. This extra period of CPR may be used to re-evaluate and summarize the diagnostic and therapeutic interventions performed and achieve the univocal team decision to terminate futile efforts. Depending on cultural and personal preferences, this period may also be used to invite family members (particularly parents of their arrested children, if not already present) to the trauma bay, allowing them to witness the resuscitation efforts and be part of the scene, when CPR is eventually terminated.

### 5.3. Exceptions from the TCA Guidelines

TCA guidelines, e.g., the ERC TCA guidelines [3], de-prioritize the immediate initiation of chest compressions and adrenaline administration, as conventionally stressed in medical cases of CA. However, there are notable exceptions, where TCA treatment should follow the standard algorithm of ALS with immediate focus on high-quality CPR with chest compression and oxygenation/ventilation. Examples are TCA after cardiac contusions, asphyxiation (e.g., burial under sand or in a crowd crush), electricity injuries, and TCA after (isolated) traumatic brain injury, caused by ‘impact brain apnea’ [60]. Thus, although there is a reshuffling of priorities of resuscitation in TCA by strictly putting forward treatment of reversible causes, this should not delay chest compressions in patients who need it. In addition, numerous trauma patients in cardiac arrest primarily suffered from a medical cause of arrest, e.g., undergoing a myocardial infarction while driving a car, followed by a car crash. It is vital that a medical cardiac arrest is not misdiagnosed as a traumatic cardiac arrest and must be treated with the universal advanced life support (ALS) algorithm. Cardiac arrest or other causes of sudden loss of consciousness (e.g., arrhythmias, hypoglycemia, stroke, and seizures) may cause a secondary traumatic event. Some observational studies have reported that ~2.5% of non-traumatic out-of-hospital cardiac arrests (OHCAs) occur in cars. For a cardiac arrest to be traumatic, an adequate mechanism of injury is required, and secondary trauma after ‘medical’ cardiac arrest is frequently not adequate. Particularly for less experienced practitioners, or practitioners usually confronted merely with trauma cases, this may be challenging, because of either a biased expectation of the case, or the usually much more impressive presentation of the trauma aspect (e.g., with wounds and blood), than of the medical emergency. In addition, medical conditions and treatment hereof (e.g., usage of anticoagulants) may greatly affect treatment and outcome of the trauma patient.

### 5.4. Adrenaline in TCA

Adrenaline is a potent vasoconstrictor, besides its cardiac effects, and thus theoretically supports vascular tone in hypovolemia. Adrenaline is the drug advocated most over the entire history of (ALS) resuscitation algorithms, and is still advocated in several current TCA algorithms (e.g., ATLS 10th edition). Although early administration of adrenaline in TCA is thus still widely taught and spread practice, the scientific foundation of its use in TCA is questionable. Retrospective studies, despite their limitations, hint towards improved survival in TCA patients given less, or no adrenaline [1,68]. Thus, with regard to an intervention as controversial as adrenaline in TCA, one should balance the ‘economics of resuscitation’ and promote higher yield interventions that could be performed by the team instead, i.e., interventions addressing potentially reversible causes of TCA. There are exceptions in which adrenaline use may be more rational, e.g., traumatic (peri-)arrest caused by spinal injuries, as described below.

### 5.5. Relative Hypovolemia in TCA

Hypovolemia in TCA is usually caused by traumatic blood loss, a prototypical form of absolute hypovolemia. In rare and thus frequently unexpected cases, relative hypovolemia may contribute to TCA. Relative hypovolemia is the condition in which the absolute blood volume may be normal, but misdistribution of blood, e.g., by vasoplegia, may cause life-threatening hypotension. In TCA, this distributive shock may be attributed to spinal cord injuries, in which sympathetic innervation of the vasculature fails (neurogenic shock). TCA by relative hypovolemia alone after spinal cord injury is exceptional, but as a complicating factor sympathetic innervation of the heart may also be impaired, aggravating the neurogenic shock to life-threatening levels, particularly in conjunction with other injuries. In contrast to absolute hypovolemia after hemorrhage, where refilling the vasculature with volume such as blood(-products) is appropriate, the vasoplegia causing relative hypovolemia (and bradycardia from cardiac denervation) may be treated with a catecholamine, e.g., adrenaline. Thus, this appears an appropriate indication for adrenaline in traumatic (peri-)arrest.

## 6. Conclusions

A paradigm shift in TCA treatment switched the traditional belief of futility of TCA resuscitation to a more optimistic perspective, at least in selected cases. Practitioners should rapidly and aggressively treat the common potentially reversible causes of TCA. Further research is needed to improve outcomes in this often young and previously healthy patient population.

## Data Availability

Not applicable.
